# OrthoVenn: a web server for genome wide comparison and annotation of orthologous clusters across multiple species

**DOI:** 10.1093/nar/gkv487

**Published:** 2015-05-11

**Authors:** Yi Wang, Devin Coleman-Derr, Guoping Chen, Yong Q. Gu

**Affiliations:** 1 USDA-ARS, Western Regional Research Center, Crop Improvement and Genetics Research Unit, Albany, CA 94710, USA; 2 Department of Plant Sciences, University of California, Davis, CA 95616, USA; 3 Bioengineering College, Campus A, Chongqing University, Chongqing 400030, China; 4 USDA-ARS, Plant Gene Expression Center, Albany, CA 94710, USA

## Abstract

Genome wide analysis of orthologous clusters is an important component of comparative genomics studies. Identifying the overlap among orthologous clusters can enable us to elucidate the function and evolution of proteins across multiple species. Here, we report a web platform named OrthoVenn that is useful for genome wide comparisons and visualization of orthologous clusters. OrthoVenn provides coverage of vertebrates, metazoa, protists, fungi, plants and bacteria for the comparison of orthologous clusters and also supports uploading of customized protein sequences from user-defined species. An interactive Venn diagram, summary counts, and functional summaries of the disjunction and intersection of clusters shared between species are displayed as part of the OrthoVenn result. OrthoVenn also includes in-depth views of the clusters using various sequence analysis tools. Furthermore, OrthoVenn identifies orthologous clusters of single copy genes and allows for a customized search of clusters of specific genes through key words or BLAST. OrthoVenn is an efficient and user-friendly web server freely accessible at http://probes.pw.usda.gov/OrthoVenn or http://aegilops.wheat.ucdavis.edu/OrthoVenn.

## INTRODUCTION

Orthologs or orthologous genes are clusters of genes in different species that originated by vertical descent from a single gene in the last common ancestor (1). Comparative analysis of the organization of orthologous clusters is important for understanding the rules of genome structure and gene/protein function. The information gained from comparisons of orthologous clusters can serve as raw material for taxonomic classification and phylogenetic studies of organisms, thereby shedding light on the mechanisms underlying the molecular evolution of genes and genomes (2,3). Recent advances in genome sequencing technologies has provided a wealth of genome sequence data from many organisms. The increasing availability of genome sequence data across the tree of life now makes it possible to conduct whole-genome comparative analyses of orthologous clusters across multiple species.

In order to identify orthologous genes from different genomes for classification within gene clusters, databases have employed different approaches that can be generally classified into two groups. One group is based on pairwise sequence comparisons (e.g. eggNOG (4), InParanoid (5), OrthoDB (6)), while the other uses phylogenetic methods (e.g. MetaPhOrs (7), PhylomeDB (8)). These analysis tools are well known and widely used, but their online servers are often database-oriented and focused on gene searches and analysis within specific orthologous groups, and lack the functionality to generate visualizations displaying the difference and overlapping for all orthologous clusters. Some of these databases also provide ortholog prediction software (InParanoid: http://software.sbc.su.se/cgi-bin/request.cgi?project=inparanoid, OrthoDB: http://www.orthodb.org/orthodb_software/), but generally the software has to be downloaded and run locally. The Quest for Orthologs Consortium (http://questfororthologs.org/) improves and standardizes orthology predictions and provides a list of >30 of these databases.

The tools for establishing homologies between genes or their products are becoming increasingly important to transfer knowledge from well-studied model organisms to other organisms (9). One of the simplest but most useful methods of genome wide orthologous comparison is to display the different and overlapping orthologous clusters in a Venn diagram, which in our case provides circles or other shapes representing each species with overlapping regions that illustrate the genes or gene clusters that are unique to or shared between each species. As an example, a recent analysis of the banana genome (http://www.promusa.org/blogpost174-The-best-genomics-Venn-diagram-ever-deconstructed), presented such a Venn diagrams for orthologous clusters comparison among six plant genomes. Venn diagrams allow for quick visualization of relationships by revealing intersections (overlaps) and disjunctions (non-overlaps) for large biological datasets obtained from different species, and are often used in the whole-genome analysis across species (10–12). Currently, a number of online Venn diagram applications have been developed to provide simultaneous visual interpretation of large amounts of biological data. The Pangloss Venn diagram generator (http://www.pangloss.com/seidel/Protocols​/venn4.cgi) and Venny (http://bioinfogp.cnb.csic.es/tools/venny​/index.html) are web applications that can create Venn diagrams from user-provided ID lists. BioVenn (13) provides a comparison and visualization of biological lists using area-proportional Venn diagrams. GeneVenn (14) and VennMaster (15) possess the additional feature of linking genes within each group to related information in the NCBI Entrez Nucleotide database or the Gene Ontology database. NetVenn (16) compares and analyzes gene lists by combining a Venn diagram visualization with an interactome network and biological annotation data. A database named EDGAR provides Venn diagrams of the common gene pools for the comparative analysis of prokaryotic genomes (17).

To our knowledge, a web application that offers genome wide comparison and analysis of orthologous clusters across multiple species is not available. SPOCS (18) provides a web server for prediction of orthologs and paralogs among closely related genomes, however, it's simple visualization function does not allow for comparison of overlapping orthologous clusters among species. Here, we present a web platform named OrthoVenn (http://probes.pw.usda.gov/OrthoVenn or http://aegilops.wheat.ucdavis.edu/OrthoVenn) for the comparison and analysis of genome wide orthologous clusters across multiple species. In OrthoVenn, users can select protein sequence data for genome wide comparisons from 272 species in the database, including vertebrates, metazoa, protists, fungi, plants and bacteria. OrthoVenn also allows user-defined species to be uploaded as customized protein sequences. An efficient and interactive graphics tool is employed to provide a Venn diagram view of the genome wide comparison of orthologous clusters based on the protein sequence data selected from up to six species. The intersection of orthologous clusters is analyzed by GO Slim annotation and UniProt search. In the output of OrthoVenn, each orthologous cluster provide sequence analysis data, single copy gene cluster identification, protein similarity comparisons, and the phylogenetic relationships among clustered genes. OrthoVenn also provides key word search and BLAST functions for finding clusters of specific interest to the user. In addition, OrthoVenn allows the user to create Venn diagrams from orthologous cluster files generated by other software.

## WEB SERVER CONSTRUCTION

### Protein sequences collection

We downloaded sequence data from the Ensembl database, which is a genomic interpretation system providing the most up-to-date annotations of whole-genome protein sequences for many species (19). After quality analysis (sequences contains illegal characters or <20 amino acids were removed) of the amino acid fasta file, we retrieved the protein sequences of all gene coding sequences without alternative splice variants. We grouped the protein sequences into six categories: vertebrates, metazoa, protists, fungi, plants and bacteria. A summary of the collected protein sequences in each category is presented in Table 1. OrthoVenn also allows user-defined protein sequences in fasta or compressed fasta (.tar.gz and .zip) format for any non-represented species. Each of these novel species can be given their own name so that the user can immediately see which part of the output in the Venn diagram corresponds to which species.

**Table 1.** Total numbers of categorized protein sequences in OrthoVenn

Category	Number of species	Number of protein sequences
Vertebrates	69	1 276 453
Metazoa	55	1 020 988
Protists	32	458 802
Fungi	52	567 086
Plants	38	1 321 298
Bacteria	26	56 830
Total	272	4 701 457

### Identification of orthologous cluster

There are multiple methods for orthology prediction. We used the popular heuristic approach named OrthoMCL (20) to identify ortholog groups. The OrthoMCL performs an all-against-all BLASTP alignment, identifies putative orthology and inparalogy relationships with the Inparanoid algorithm (21) and generates disjoint clusters of closely related proteins with the Markov Clustering Algorithm (MCL) (22). The steps in the OrthoMCL are time-consuming for large numbers of protein sequences from multiple species. In order to make the approach suitable for the web application, we modified portions of the data processing steps in the OrthoMCL algorithm. First, we used UBLAST (v7.0.1090) (23) to do the all-against-all similarity search. UBLAST is ∼350× faster than BLASTP and achieves very similar results for ortholog searches (24,25). Second, each of the protein sequences downloaded from Ensembl have been aligned with one another and the result is stored in the server as BLAST tab format data (∼1.6TB). If the input species for a new OrthoVenn analysis is contained in the previously sorted alignment table, the step for sequence comparison will be omitted in the process. Finally, we used orthAgogue (v1.0.3) for identification of putative orthology and inparalogy relations. The orthAgogue is a multithreaded C application for high-speed estimation of homology relations in massive datasets (26). We have compared the orthologous clustering results between OrthoMCL and OrthoVenn, and they have similar clustering behavior (Supplemental Material).

### Orthologous cluster annotation

To deduce the putative function of each ortholog, the first protein sequence in each cluster are subjected to BLASTP analysis against the non-redundant protein database in UniProt (27). We only selected one sequence in each cluster because the proteins are very similar and performing BLASTP on all sequences in each cluster against UniProt is computationally too expensive, especially for large genomes which contain many protein sequences. Any hit with an e-value <1e−5 is considered and the top hit is defined as the putative function of each cluster. The GOSlim terms for biological process, molecular function, and cellular component categories are assigned to the corresponding orthologous cluster. The GOSlimViewer (28) is used to provide a high-level summary of functions for orthologous clusters in the Venn diagram overlapping regions using pre-computed GO Slim sets. An alignment of the protein sequences in each cluster is performed using the multiple sequence alignment tool MUSCLE (29). The conserved motifs in the protein sequences are identified by Multiple Expectation Maximization for Motif Elicitation program (MEME) (30). Phylogenetic trees for the orthologous clusters are built in FastTree (31).

### Implementation

OrthoVenn was implemented with a Java code as the back end, configured on a Ubuntu Linux with an Apache server (http://www.apache.org/). PHP 5.5, JavaScript and HTML were used to develop the front-end user interface. The user input is sent to a PHP script through an interactive form and calls a core program written by Java. After the program is finished running, the PHP script retrieves and displays the results with hyperlinks to additional information. The Venn diagram is presented through jvenn, which is a new JavaScript library that allows the developer to embed Venn diagram viewers within HTML documents (32). Since we expect that the protein sequences in the Ensembl database will be updated regularly, we will update the OrthoVenn dataset every half year with our semi-automatic sequence identification pipeline.

## SAMPLE AND RESULT ANALYSIS

We applied OrthoVenn clustering to identify gene clusters enriched in six grass genomes including *Aegilops tauschii* (Tausch's goatgrass), *Brachypodium distachyon* (false brome), *Oryza sativa* (rice), *Sorghum bicolor* (sorghum), *Hordeum vulgare* (Barley) and *Zea mays* (maize). Pairwise sequence similarities between all input protein sequences were calculated with an e-value cut-off of 1e−5. An inflation value (−*I*) of 1.5 was used to define orthologous cluster structure. The result is present at http://probes.pw.usda.gov/OrthoVenn/result.php?ID=g5.

**Figure 1.**
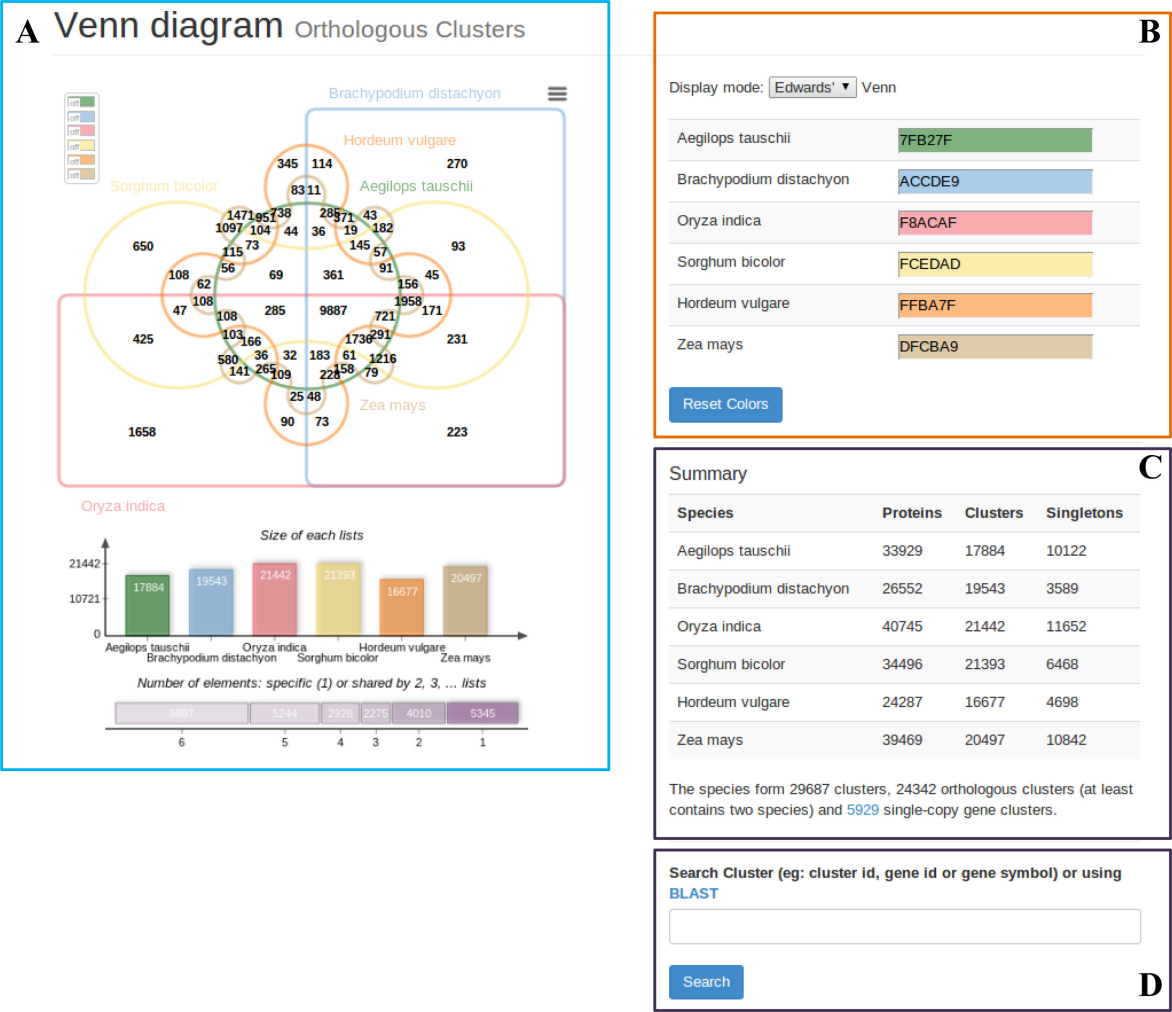
A results page in OrthoVenn. (**A**) Venn diagram showing the distribution of shared gene families (orthologous clusters) among *Aegilops tauschii, Brachypodium distachyon, Oryza sativa, Sorghum bicolor, Hordeum vulgare* and *Zea mays*. The cluster number in each component is listed. (**B**) Color selector and display mode setting for the Venn diagram. (**C**) Counts of clusters in each genome. (**D**) Key word search and BLAST links for finding specific clusters in the result.

**Figure 2.**
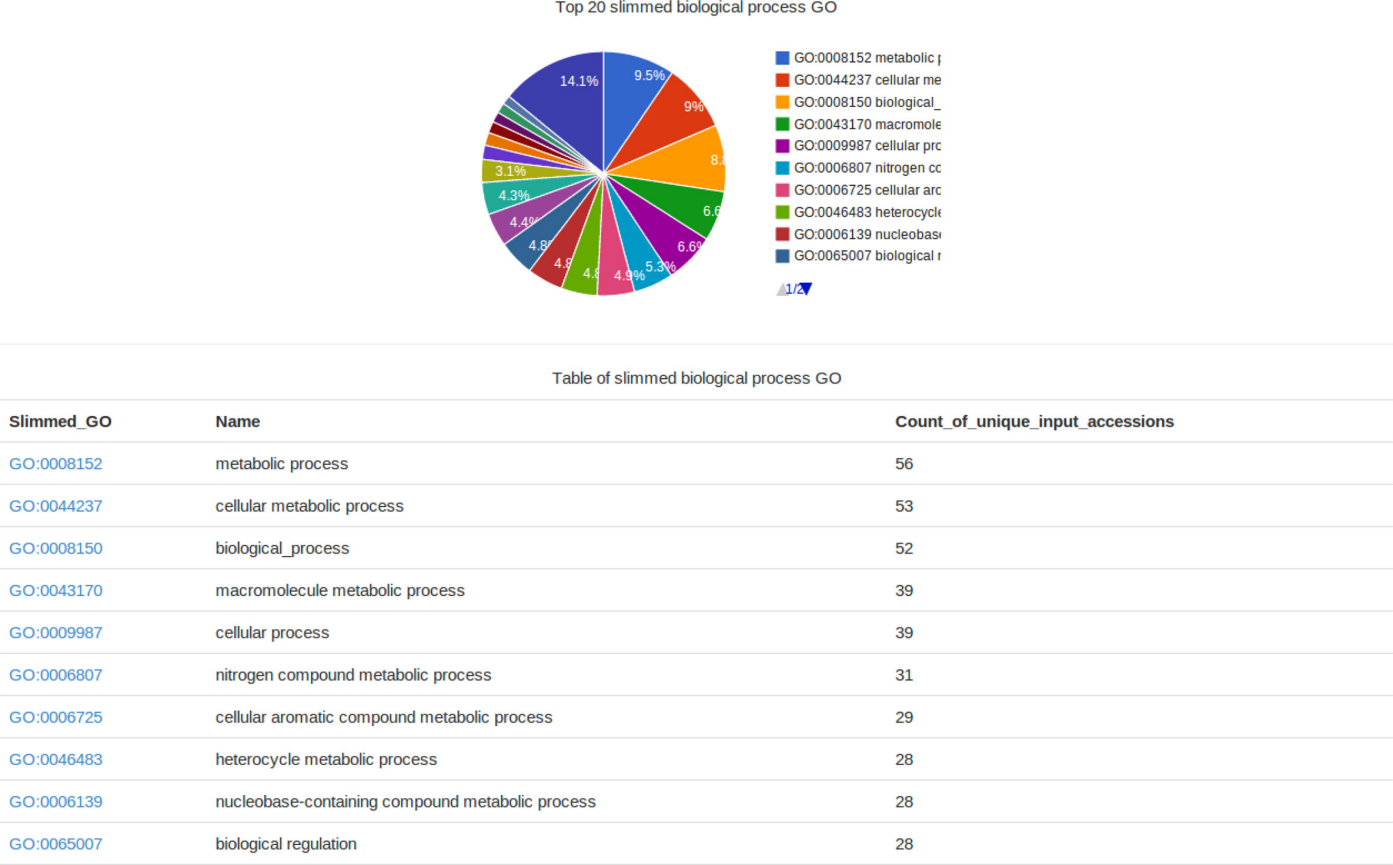
Distributions of *Aegilops tauschii* specific gene sets in biological process GO slim terms.

Four main components are shown in the results page: a Venn diagram showing the shared orthologous gene clusters among six grass genomes (Figure 1A), a control panel for setting the colors and Venn diagram display mode (Figure 1B), a table of the number of clusters in each genome (Figure 1C), and the key words input and BLAST link for performing user-defined searches (Figure 1D). The analysis showed that 24 342 orthologous clusters were formed based on the protein sequences from the 6 species. These observations are consistent with that reported in a recent study which found 23 202 orthologous groups in five grass genomes (33). The numbers in the Venn diagram represent the number of orthologous clusters that *A. tauschii* shares with the five other species. The diagram shows that 9887 gene clusters are shared by all six species, suggesting their conservation in the lineage after speciation. Additionally, it shows that there were 951 clusters specific to *A. tauschii*. These clusters are likely gene clusters within multiple genes or in-paralog clusters. The presence of the in-paralog clusters suggests that there might be a lineage specific gene expansion in these gene families in *A. tauschii*. Based on annotation of these clusters, some of these lineage specific clusters could be potentially involved in important biological processes (e.g. nitrogen compound metabolic process or cellular aromatic compound metabolic process) (Figure 2).

**Figure 3.**
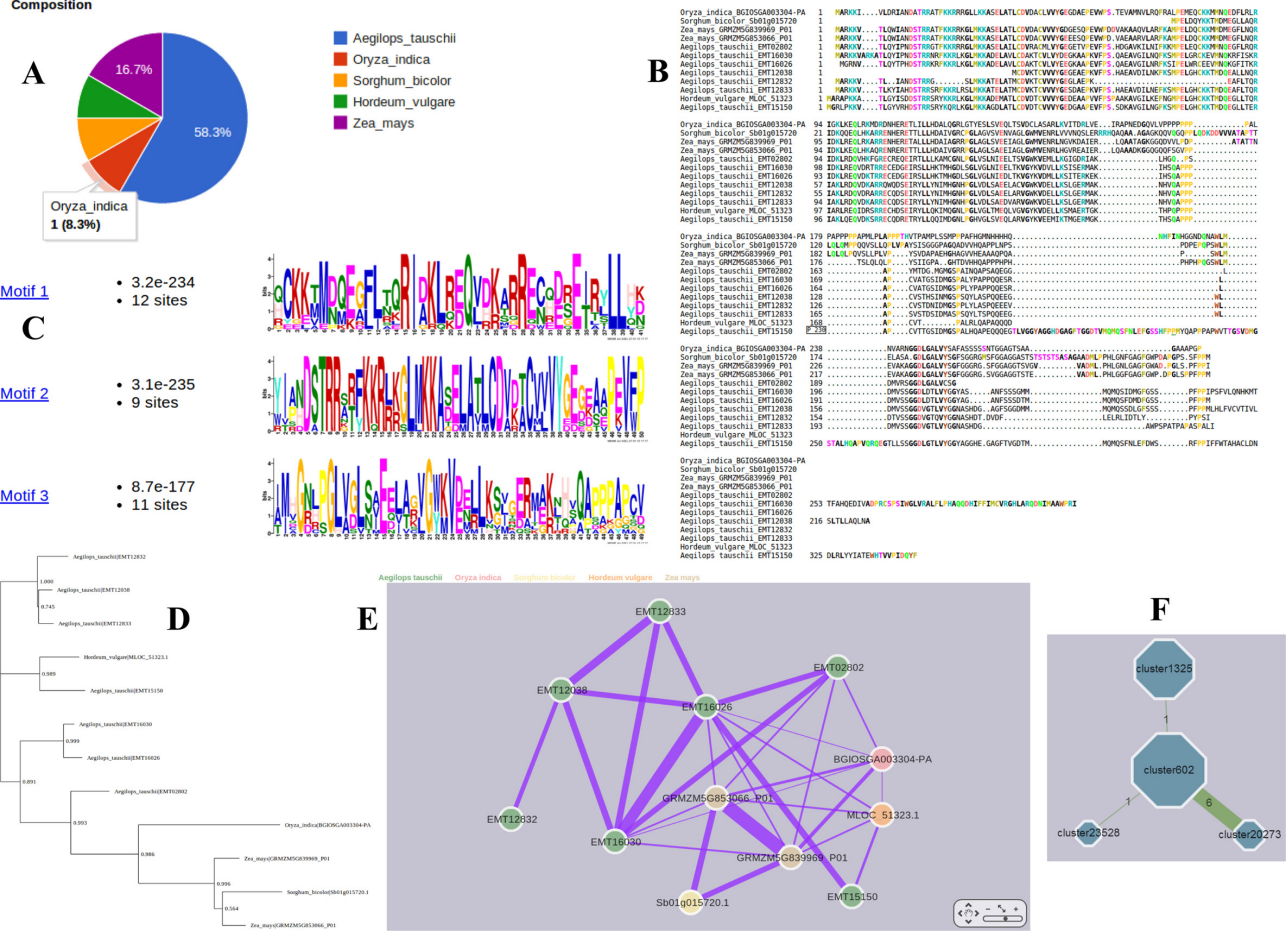
The annotation of cluster602 with different methods. (**A**) Composition of the cluster. (**B**) Multiple sequence alignment viewer. (**C**) Motifs in the proteins of the cluster. (**D**) Phylogenetic tree showing the inferred evolutionary relationships among the sequences in cluster602. (**E**) Network layout of the cluster. Nodes represent proteins and the edge width indicates the similarity between protein nodes. (**F**) The relationships between cluster602 and other clusters. Each node is a cluster and node size represents the number of proteins in the cluster. The edge weight means the amount of similar sequences by counting similar sequence pairs between the clusters.

The identification of single copy orthologs in any group of species is important for phylogenetic studies (34). Based on the orthologous clustering result, we have identified a set of 5929 clusters representing single copy genes shared in all six genomes, suggesting they maintained single copy status through evolutionary time after the divergence of these species. Each orthologous cluster in the OrthoVenn result provides an in-depth view of the genes and their sequences (Figure 3). Users can search specific clusters using key words, however, a BLAST search function is also provided. In the case of the key word search, input of ‘Agamous-like MADS-box’ will result in an orthologous cluster search result. Selection of cluster602 from the search result indicates that this orthologous group contains 12 protein sequences from five species (Figure 3A). Multiple sequence alignment and motif analysis indicate that the cluster represents a typical MADS-box protein (Figure 3B and C). Phylogenetic tree data shows that in cluster602, *A. tauschii* contains more paralogous genes, suggesting there is lineage-specific expansion of this specific type of MADS-box protein (Figure 3D). Furthermore, OrthoVenn includes the Cytoscape Web application (35) for visualizing and manipulating the graphs of the cluster and the relationship between clusters. In cluster602, proteins from the same species had higher similarity to one another than proteins between different species (Figure 3E), suggesting that duplication events after speciation resulted in closely related paralogous genes. In addition, cluster602 is closely related to cluster20273, cluster1325 and cluster23528, as revealed by the width of the lines connecting the cluster nodes (Figure 3F). Related clusters are defined by sequence similarity of proteins in these clusters as determined by the orthAgogue analysis result. The edge weight means the amount of similar sequences by counting similar sequence pairs between the clusters. Annotation data indicates that all of these clusters are Agamous-like MADS-box proteins.

## VENN DIAGRAM GENERATION FROM CLUSTER FILE

In order to display orthologous cluster files directly in a Venn diagram, we developed a tool named ClusterVenn in the web site of OrthoVenn for converting clusters file to Venn diagrams. This allows users to submit their own orthologous cluster files generated by other software such as OrthoMCL. ClusterVenn calculates the number of species in the file, and provides a convenient way for users to choose which species should be compared. ClusterVenn then identifies the shared and unshared orthologous clusters based on identifiers in the orthologous cluster file and displays the Venn diagram as the result.

## DISCUSSION

With the rapid increase of genome sequence data from high-throughput sequencing technologies, effective bioinformatics tools for genome wide comparisons among species has become an important component of gene and genome analysis. The identification of orthologs among species is a fundamental step in many comparative genomics studies. Visualization of disjointed or intersecting orthologous clusters provides insights into genome evolution across multiple species. OrthoVenn is a unique web tool for the visualization of genome wide comparisons of orthologous clusters with an interactive Venn diagram view and provides a high-level summary of functions for overlapping and non-overlapping orthologous gene sets. The integration of several sequence analysis methods in OrthoVenn provides further information related to the sequence conservation of orthologous genes of interest to the user. Furthermore, OrthoVenn is an easy-to-use web server that only requires users to choose species from the available list of 272 organisms or to submit a list of protein sequences for their species of interest, and allows the ortholog analysis to be performed without the need to install programs locally. We think OrthoVenn provides a powerful platform for identifying, analyzing and assigning the biological meaning to orthologous genes and facilitates a better understanding of gene and genome evolution across diverse taxa.

## AVAILABILITY

http://probes.pw.usda.gov/OrthoVenn or http://aegilops.wheat.ucdavis.edu/OrthoVenn.

## SUPPLEMENTARY DATA

Supplementary Data are available at NAR Online.

SUPPLEMENTARY DATA
