# Supplementary material for: OrthoVenn: a web server for genome wide comparison and annotation of orthologous clusters across multiple species
Source: Nucleic Acids Res. 2015 May 11;43(Web Server issue):W78–84. doi: 10.1093/nar/gkv487 (PMC4489293; doi:10.1093/nar/gkv487)
Supplement: SUPPLEMENTARY DATA [file supp_43_W1_W78__index.html]

OrthoVenn: a web server for genome wide comparison and annotation of orthologous clusters across multiple species — SUPPLEMENTARY DATA 

# OrthoVenn: a web server for genome wide comparison and annotation of orthologous clusters across multiple species

## SUPPLEMENTARY DATA

- SUPPLEMENTARY DATA
