# Supplementary material for: OrthoVenn: a web server for genome wide comparison and annotation of orthologous clusters across multiple species
Source: Nucleic Acids Res. 2015 May 11;43(Web Server issue):W78–84. doi: 10.1093/nar/gkv487 (PMC4489293; doi:10.1093/nar/gkv487)
Supplement: SUPPLEMENTARY DATA [file supp_gkv487_nar-00371-web-b-2015-File003.pptx]

## Slide 1
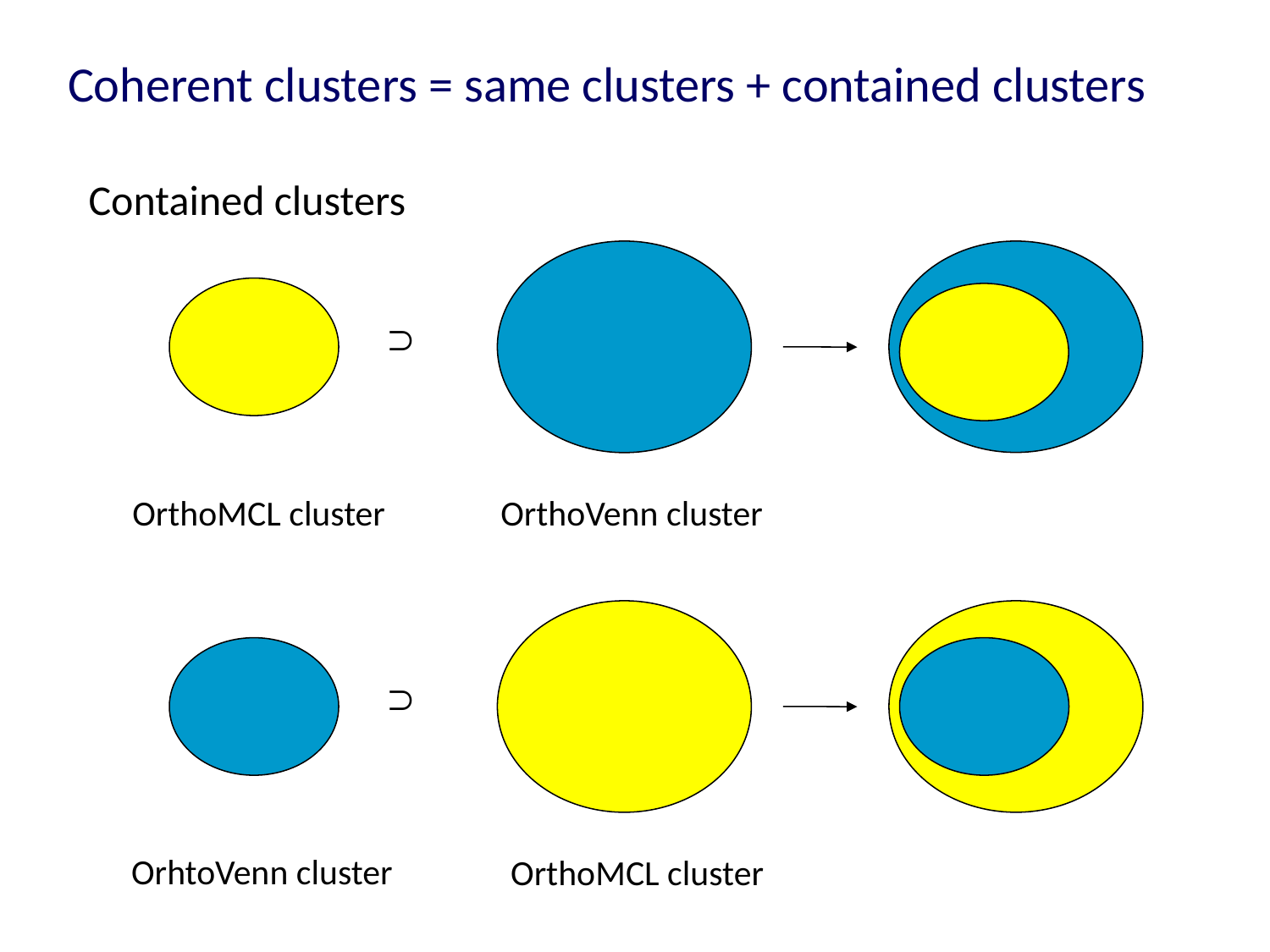

Coherent clusters = same clusters + contained clusters
Contained clusters
∩
OrthoMCL cluster
OrthoVenn cluster
∩
OrhtoVenn cluster
OrthoMCL cluster

## Slide 2
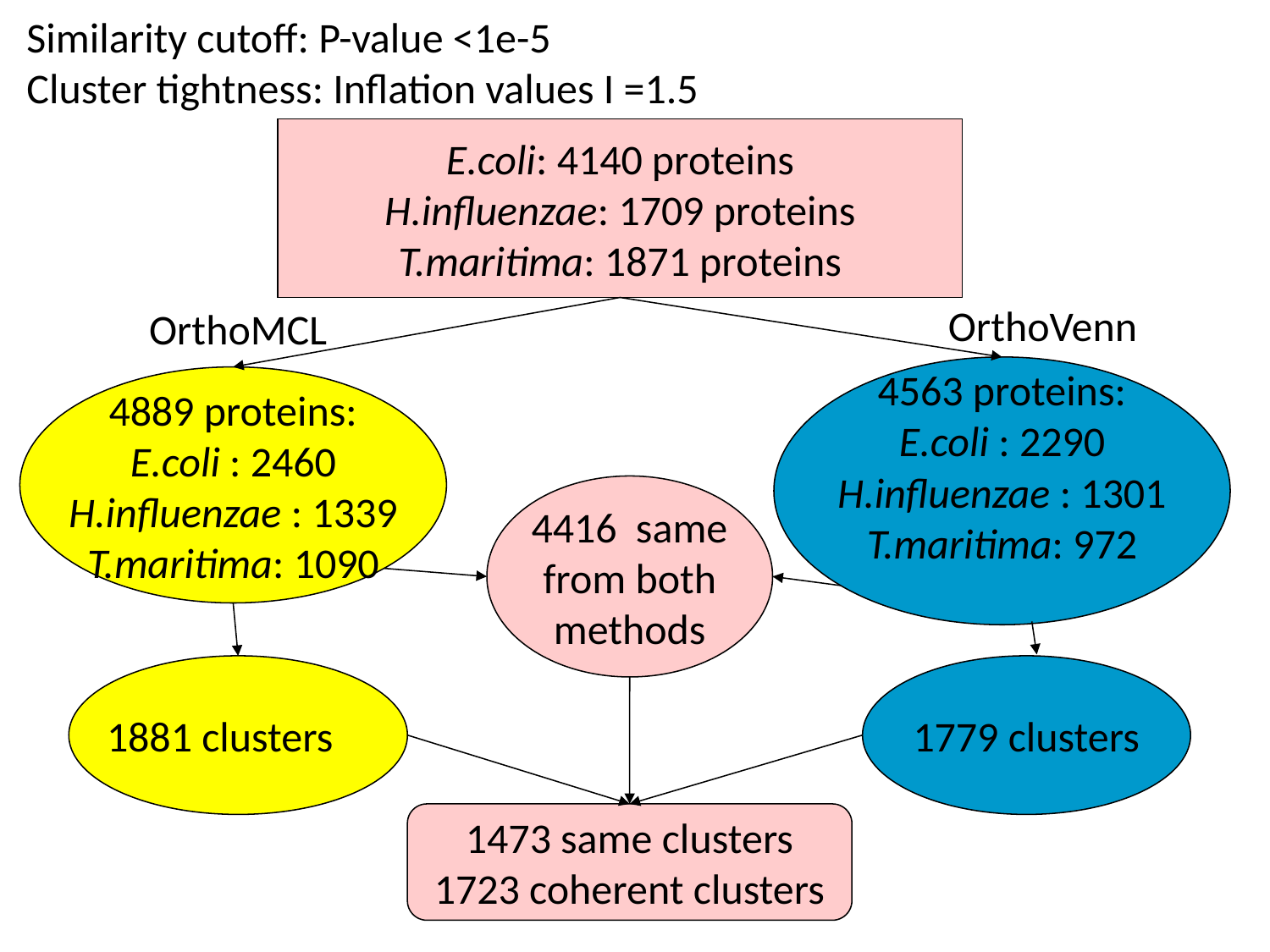

Similarity cutoff: P-value <1e-5
Cluster tightness: Inflation values I =1.5
E.coli: 4140 proteins
H.influenzae: 1709 proteins
T.maritima: 1871 proteins
OrthoVenn
OrthoMCL
4563 proteins:
E.coli : 2290
H.influenzae : 1301
T.maritima: 972
4889 proteins:
E.coli : 2460
H.influenzae : 1339
T.maritima: 1090
4416 same
 from both
methods
1779 clusters
 1881 clusters
1473 same clusters
1723 coherent clusters

## Slide 3
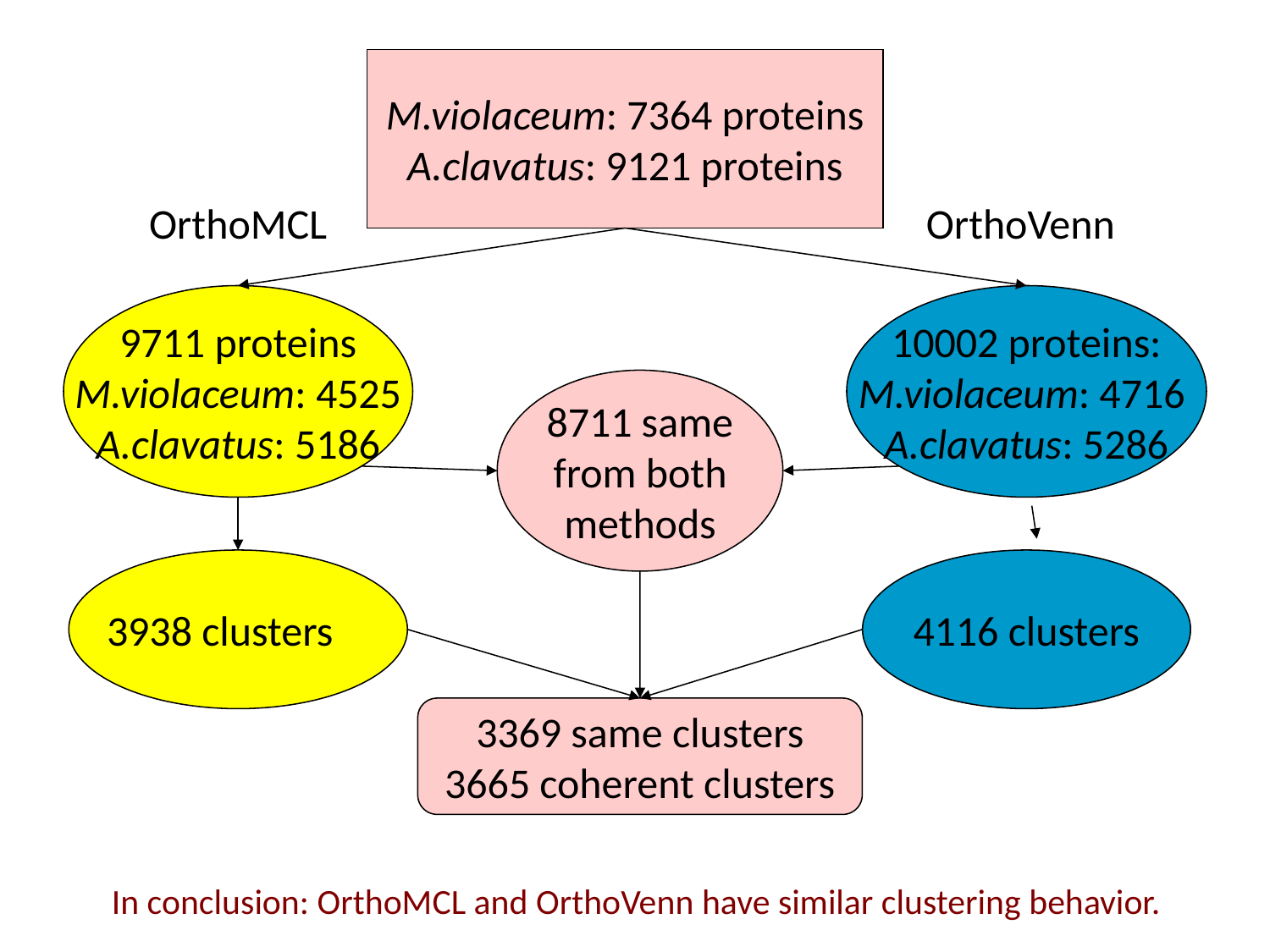

M.violaceum: 7364 proteins
A.clavatus: 9121 proteins
OrthoMCL
OrthoVenn
9711 proteins
M.violaceum: 4525
A.clavatus: 5186
10002 proteins:
M.violaceum: 4716
A.clavatus: 5286
8711 same
 from both
methods
4116 clusters
 3938 clusters
3369 same clusters
3665 coherent clusters
In conclusion: OrthoMCL and OrthoVenn have similar clustering behavior.
